# Predicting knee pain severity in professional distance runners using gaussian process regression

**DOI:** 10.3389/fpubh.2026.1973629

**Published:** 2026-10-27

**Authors:** Yunus Sadır, Mehmet İnan, Mustafa Karadağ, Levent Ceylan, Tülay Ceylan, Heidar Sajedi, Faik Öz, Yunus Şahinler, Mohammad Alimoradi, Mohammad Alghosi, Andreas Konrad

**Affiliations:** 1 Pasinler Atatürk Regional Boarding Middle School, Erzurum Provincial Directorate of National Education, Ministry of National Education (Turkey), Erzurum, Türkiye; 2 Department of Coaching Education, Faculty of Sports Sciences, Istanbul Rumeli University, Istanbul, Türkiye; 3 Department of Coaching Education, Faculty of Sports Sciences, Fırat University, Elazığ, Türkiye; 4 Department of Sport Management, Faculty of Sports Sciences, Hitit University, Çorum, Türkiye; 5 Çorum Provincial Directorate of National Education, Ministry of National Education (Turkey), Çorum, Türkiye; 6 Department of Coaching Education, Faculty of Sport Sciences, Istanbul Esenyurt University, Istanbul, Türkiye; 7 Department of Movement and Training Sciences, Faculty of Sports Sciences, Düzce University, Düzce, Türkiye; 8 Department of Exercise and Sport Sciences, Faculty of Sport Sciences, Avrasya University, Trabzon, Türkiye; 9 Red and River Digital Publishing and Trade Limited Company, Rize, Türkiye; 10 Department of Exercise and Sports Sciences for Individuals with Disabilities, Faculty of Sports Sciences, Istanbul Gelişim University, Istanbul, Türkiye; 11 Department of Sports Injuries and Corrective Exercises, Faculty of Sports Sciences, Shahid Bahonar University of Kerman, Kerman, Iran; 12 HERC – Health, Exercise and Research Center, Dubai, United Arab Emirates; 13 Department of Physical Education, Technical and Vocational University (TVU), Tehran, Iran; 14 Institute of Human Movement Science, Sport and Health, University of Graz, Graz, Austria

**Keywords:** Gaussian process regression, knee pain analysis, pain-based continuous risk score, professional distance male runners, resistance training frequency, training load

## Abstract

**Background:**

Knee pain is common in runners and may precede injury; clinical labels are sparse, but repeated symptom monitoring is practical for training decisions. The purpose of this paper is to develop a probabilistic, interpretable model that predicts a continuous knee pain severity score in professional distance runners using Gaussian Process Regression (GPR), and to quantify the relative influence of routinely measurable training and anthropometric variables.

**Methods:**

Fifty professional male distance runners aged 20–30 years were recruited from professional athletics in Ankara, Turkey. Six predictors were used: resistance training frequency, weekly running distance, training surface, running technique (foot-strike category), history of prior knee problems, and body mass index (BMI). The outcome was a continuous knee pain severity score normalized to 0–1 from a self-reported numerical rating scale. A GPR model with a radial basis function kernel was trained using a train-test split and evaluated with mean squared error (MSE).

**Results:**

Prediction uncertainty was quantified using 95% credible intervals, and sensitivity analysis were performed to assess feature influence. The GPR model achieved low prediction error on the held-out test set [MSE = (0.02)] and stable performance under cross-validation [mean MSE = (0.028)].

**Conclusion:**

Sensitivity analysis showed pronounced GPR response patterns for resistance training frequency and BMI, with smaller response patterns for weekly running distance and prior knee problems/injuries. Training surface and running technique showed comparatively limited sensitivity. GPR effectively models self-reported pain with uncertainty quantification and is scalable for symptom-based risk assessment, though not intended for clinical diagnosis.

## 1 Introduction

Knee-related problems are a common concern in distance running, as repetitive lower-limb loading exposes the knee to high cumulative stress and is associated with a substantial burden of knee-related complaints and long-term knee health in runners (1, 2). Frequently reported conditions include patellofemoral pain, iliotibial band–related symptoms, and patellar tendinopathy, which can lead to symptom persistence, modified training, or temporary withdrawal from training and competition (2, 3). The prevalence of knee symptoms and related time-loss episodes highlights the need for practical monitoring and prevention approaches tailored to endurance athletes.

Multiple intrinsic and extrinsic factors have been associated with knee symptom development and overuse problems in runners. These include training load characteristics such as intensity and weekly volume (4, 5), biomechanical and technique-related factors (e.g., foot-strike pattern and footwear-related considerations) (6), the presence or absence of resistance training as part of the conditioning program (7–9), and athlete-specific characteristics such as body mass index (BMI) (10, 11) and history of previous knee problems/injuries (12). Importantly, these factors often interact in complex and non-linear ways, making it difficult to translate isolated risk factors into individualized estimates of future symptom burden. As a result, there is growing interest in data-driven approaches that can integrate multiple predictors simultaneously.

Machine learning (ML) methods have been increasingly adopted in sports science as tools to model complex relationships between training frequency, athlete characteristics, and health outcomes (13–15). ML approaches are commonly categorized into unsupervised and supervised techniques. Unsupervised methods are useful for discovering patterns or clustering movement strategies without labeled outcomes, and they have been applied to areas such as technique characterization and biomechanical monitoring (16–20). However, when the goal is to predict an outcome for a given athlete (e.g., symptom severity), supervised learning is generally required because it learns mappings from labeled input–output data. Supervised ML methods such as decision trees and support vector machines have been used in athlete monitoring contexts because they can capture non-linear relationships and handle multi-factorial inputs (21, 22). In this study, we used Gaussian Process Regression (GPR) to model a continuous, normalized knee pain severity score in professional distance runners. GPR is a non-parametric supervised learning approach well suited to small-to-moderate datasets and is distinguished by its ability to provide both predictions and principled uncertainty estimates (23). Unlike many deterministic regression models, GPR can flexibly capture nonlinear relationships while quantifying predictive uncertainty, which may be particularly useful for athlete monitoring settings where measurements are imperfect and sample sizes are constrained (24, 25).

The primary objective of this study is to develop and evaluate a GPR-based framework to predict an individualized self-reported knee pain severity score in professional distance runners aged 20–30 years using routinely obtainable predictors (resistance training frequency, weekly running distance, training surface, running technique, history of previous knee problems/injuries, and BMI). We hypothesize that (i) GPR can achieve low prediction error for knee pain severity in this cohort, and (ii) sensitivity and interaction analyses will highlight nonlinear effects and combinations of predictors associated with higher predicted pain severity. The proposed model is intended as a probabilistic, symptom-based decision-support tool and should not be interpreted as prediction of clinically diagnosed injury.

## 2 Materials and methods

As outlined in the introduction, in this paper, we focus on the problem of predicting knee pain levels of professional male runners aged 20–30. The goal is to introduce surrogate machine learning platform that is capable of accurate and fast predictions along with the corresponding uncertainties. In the following, we will present our experiments to generate dataset suitable for machine learning trainings, then, we will propose briefly the GPR technique that is utilized for our data training purposes.

### 2.1 Study design and participants

This study recruited professional male distance runners from professional athletics clubs in Ankara, Turkey using convenience sampling. “Professional distance runner” was defined as: officially registered with a national athletics federation, ≥5 years of systematic endurance training, training ≥6 days/week, and weekly running volume exceeding 100 km with regular participation in national-level or regional competitive events.

Inclusion criteria: male, 20–30 years, meeting the professional definition above, and actively training at the time of data collection.

Exclusion criteria: acute injury preventing training at baseline, recent surgery, or conditions limiting participation.

### 2.2 Ethics and consent

Ethical approval was obtained from the Ethics Committee of the International University of Science and Technology (Approval No: E-4651465861-204.01.07-195). The University operates as an international institution with a branch campus in Ankara, Turkey, and the present data collection was conducted through this Ankara branch in collaboration with local athletics clubs. Written and verbal informed consent was obtained from all participants prior to participation, and all data were anonymized before analysis. The study was conducted in accordance with the Declaration of Helsinki and applicable institutional and national requirements.

### 2.3 Measures and dataset

A dataset of 50 samples was created. Six predictors were collected via structured questionnaire and assessment, as shown in Figure 1:

1. Resistance training frequency (RTF): Resistance-training frequency was recorded over the preceding 4 weeks as a five-level ordinal variable (1–5), with higher scores representing greater typical weekly resistance-training frequency.
2. Weekly running distance (km/week): self-reported typical weekly mileage over the last 4 weeks.
3. Training surface: Surface type was categorized using a dominant-surface rule based on the preceding 4 weeks. If ≥60% of weekly distance was performed on one surface type, that surface was coded as dominant; otherwise the athlete was coded as mixed. Coding was: 1 = predominantly soft (grass/tartan/trails), 2 = mixed, 3 = predominantly hard (asphalt/concrete). For example, an athlete running ~60% on asphalt, 20% tartan, 20% trails would be categorized as predominantly hard (3).
4. Running technique: coded as a binary nominal foot-strike category (assessed by coach observation and self-report):

- 2 = midfoot strike
- 3 = heel strike

1. Prior knee problems/injuries: count of prior knee issues within previous 12 months. An “episode” was defined as knee pain or injury that resulted in a modified training ≥7 days and/or medical consultation. The variable was included as an integer count.
2. Body mass index (BMI): kg/m^2^, measured using Tanita scale outputs for mass and measured height.

**Figure 1:**
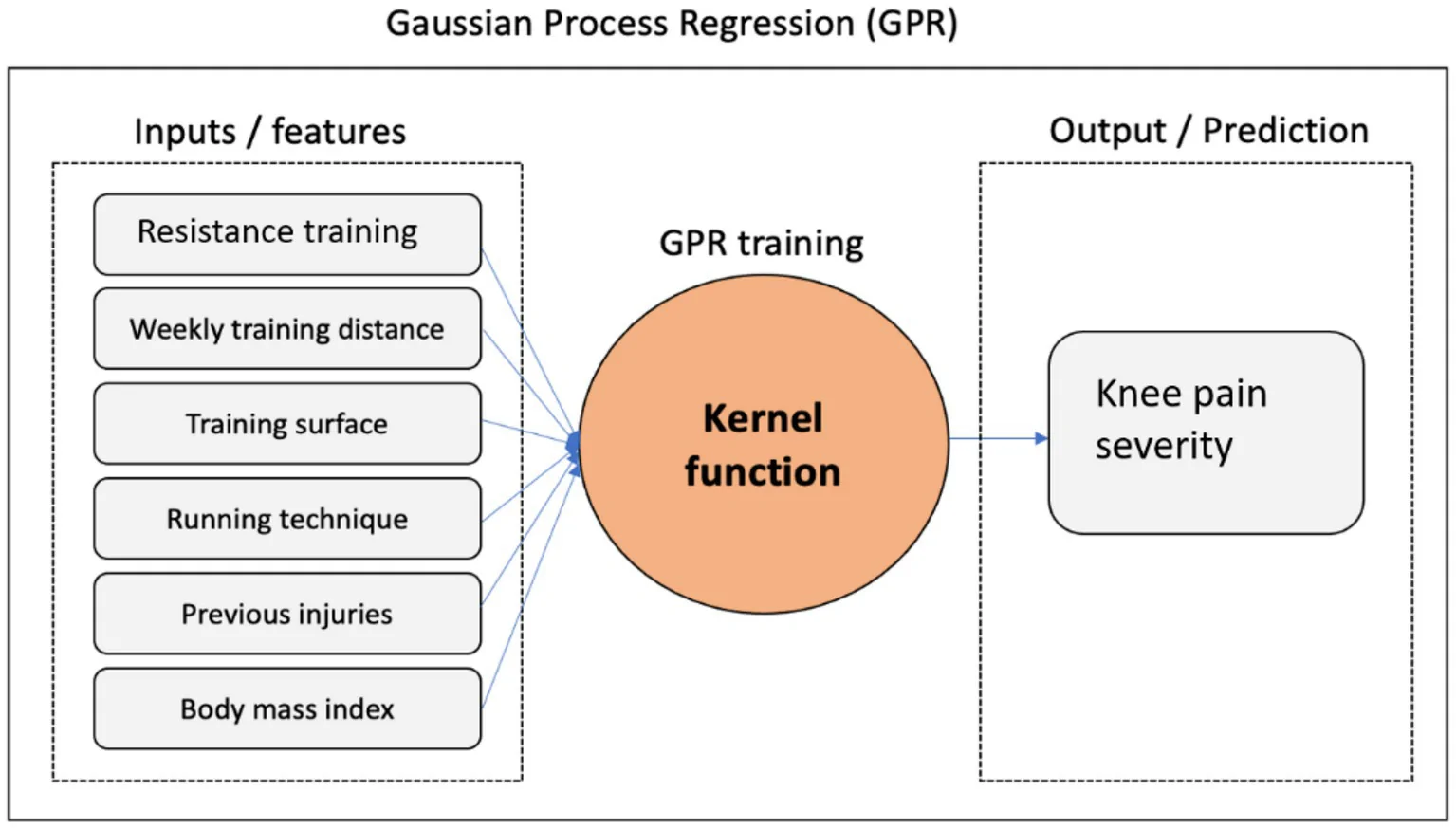
Schematic presentation of the GPR training for predicting knee pain severity with six input features.

Resistance training frequency and training surface were treated as ordinal variables, with training surface reflecting increasing surface hardness, whereas running technique was represented as a binary nominal variable. Because numerical encoding within an RBF kernel can impose artificial distances between categories, alternative categorical encoding was also examined as a sensitivity analysis; this did not materially alter model performance or the overall conclusions.

The outcome variable was a continuous self-reported knee pain severity score, not a clinician-confirmed injury diagnosis. Athletes rated knee pain severity during training/competition using a numerical scale of 0–10. Scores were normalized to the 0–1 range for modeling. This measure reflects perceived knee-related discomfort and functional limitation. The model should be interpreted as symptom-based risk modeling, not injury diagnosis.

### 2.4 Gaussian process regression design and implementation

Given the relatively small sample size, Gaussian Process Regression (GPR) was selected due to its suitability for small datasets and built-in regularization through kernel-based modeling. GPR is a powerful probabilistic ML technique that is particularly well-suited for regression tasks, including predicting continuous outcomes such as injury likelihood in sports science (26). The GPR has also been applied successfully in other fields, including engineering (27), material science (28), image processing (29), and medicine and healthcare (30–32). Unlike traditional regression methods that assume a parametric form for the relationship between inputs and outputs, GPR treats the predictions as a distribution over functions. This flexibility allows GPR to capture complex relationships in the data without imposing rigid assumptions about the underlying functional form.

### 2.5 Gaussian process

The main idea behind GPR is based on Gaussian processes, which define distributions over functions where any finite set of points have a joint Gaussian distribution (33). In GPR, we assume a Gaussian prior over functions and update this prior based on the observed data to obtain a Gaussian posterior distribution. For a set of input data points $X=[x_{1},x_{2},\ldots ,x_{n}]$ and corresponding output values $y=[y_{1},y_{2},\ldots ,y_{n}]$ , we assume that the function values $f(X)=[f(x_{1}),f(x_{2}),\ldots ,f(x_{n})]$ are jointly Gaussian.The mathematical formulation of the GPR model is presented in Equations 1–12:

f(X)=N(μ(X),K(X,X)) (1)

Where μ(X) is the mean function, typically assume to be zero μ(X)=0 without loss of generality, and K(X,X) is the covariance matrix define by the kernel function.

For a new input or test point $x_{*}$ , the predictive distribution is also Gaussian:

$$p(y_{*}|,x_{*}|,X|,y)=N(\mu_{*},\sigma_{*}^{2}\;)$$ (2)

Where $\mu_{*}$ is the mean prediction and $\sigma_{*}^{2}$ is the variance, which quantify the uncertainty in the prediction (34). The predictive mean and variance can be computed as:

$$\mu_{*}=k{\left(x_{*},X\right)}^{T}{\left(K(X,X)+\sigma_{n}^{2}I\right)}^{-1}y\;$$ (3)

$$\sigma_{*}^{2}=k(x_{*},x_{*})-k{\left(x_{*},X\right)}^{T}{\left(K(X,X)+\sigma_{n}^{2}I\right)}^{-1}k(x_{*},X)$$ (4)

Where $k{\left(x_{*},X\right)}^{T}$ is the covariance vector between the new point $x_{*}$ and the training points, K(X,X) is the covariance matrix of the points, and $\sigma_{*}^{2}$ represents the noise variance.

### 2.6 Covariance functions (kernels)

In practice, GPR involves choosing a suitable kernel function (or covariance function) that reflects the underlying structure of the data. Popular choices include the Radial Basis Function (RBF) kernel and the Matern kernel, each offering different degrees of smoothness and flexibility in capturing data patterns (23). In this paper, we use the RBF kernel which is denoted as:

$$k(x_{i},x_{j})=\sigma_{f}^{2}\exp(-\frac{1}{2l^{2}}{\left(x_{i}-x_{j}\right)}^{T}(x_{i}-x_{j}))$$ (5)

Where $\sigma_{f}^{2}\;$ controls the vertical variation amplitude (variance) of the function, and l (length scale) controls the smoothness of the function.

For multiple inputs, the covariance matrix K(X,X) is constructed with entries $K_{\mathrm{ij}}=k(x_{i},x_{j})$ , leading to the full covariance matrix:

$$K(X,X)=[\begin{array}{l} k(x_{1},x_{1})\;k(x_{2},x_{1})\vdots k(x_{n},x_{1})\;\;k(x_{1},x_{2})\;k(x_{2},x_{2})\\ \vdots k(x_{n},x_{2})\cdots \cdots \ddots \cdots \;\;k(x_{1},x_{n})\;k(x_{2},x_{n})\vdots k(x_{n},x_{n}) \end{array}]$$ (6)

### 2.7 Training the GPR model

In the GPR, the model is trained by optimizing the hyperparameters of the kernel function and maximizing the log marginal likelihood of the observed data D={(X,y)} , ensuring the best fit of the Gaussian process to the training dataset. The log marginal likelihood logp(y∣,X∣,θ) given hyperparameters θ is given by:

$$\log\;p(X,\theta )=-\frac{1}{2}y^{T}{\left(K+\sigma_{n}^{2}I\right)}^{-1}y-\frac{1}{2}\text{loglog}\;|K+\sigma_{n}^{2}I|-\frac{n}{2}\log(2\pi )$$ (7)

Here, K is the covariance matrix with entities determined by the kernel function, $\sigma_{n}^{2}\;$ is the noise parameter, and n is the number of data points.

The dataset was randomly divided into a training set (70%) and an independent test set (30%) using a fixed random seed. Hyperparameter optimization was performed exclusively within the training set using 5-fold cross-validation and GridSearchCV based on mean squared error. The optimized GPR model was subsequently fitted to the training data and evaluated on the held-out test set, which was not used during model training or hyperparameter selection. In addition, 5-fold cross-validation was performed as a complementary assessment of model stability.

GPR’s probabilistic nature provides several advantages in pain prediction for professional distance runners. It inherently quantifies uncertainty in predictions (34), crucial for assessing the reliability of pain related risk estimates. Moreover, GPR accommodates nonlinear relationships and can incorporate prior knowledge effectively, making it suitable for integrating diverse sources of information such as biomechanical factors, training history, and individual characteristics. This capability enhances the robustness of pain level prediction models, offering valuable insights into preventive strategies tailored to the specific needs of athletes.

### 2.8 Posterior distribution

Once the hyperparameters are optimized, the posterior distribution of the function values given the training data can be written as:

$$f_{*}\;|\;X,y,x_{*}\sim N(f_{*}\;|,\;\mu_{*},\sigma_{*}^{2}\;)$$ (8)

The posterior distribution defined above provides not only a point estimate but also an uncertainty measure, which is particularly valuable in pain level prediction.

### 2.9 Noise model and likelihood

The GPR model can incorporate a noise model to account for observation noise in the data. Assuming additive Gaussian noise with variance $\sigma_{n}^{2}$ , the likelihood of the observations given the latent function values becomes:

$$p(y\;|\;f)=N(y\;|\;f,\sigma_{n}^{2}I)$$ (9)

The marginal likelihood of the observed data can be expressed as:

p(y∣X,θ)=∫p(y∣f)p(f∣X,θ)df (10)

Which leads to the previously stated log marginal likelihood expression.

### 2.10 Predictive variance and confidence intervals

The predictive variance $\sigma_{*}^{2}$ allows us to create confidence intervals for predictions. A 95% confidence interval for the prediction at new points $x_{*}$ can be calculated as:

$$\left[\mu_{*}-1.95\cdot \sigma_{*},\mu_{*}+1.95\cdot \sigma_{*}\right]$$ (11)

We can use the above confidence level to predict different pain scenarios, where understanding the range of possible outcomes is crucial for the making appropriate decisions.

### 2.11 Sensitivity analysis and gradients

We can also carry out sensitivity analysis with respect to inputs by computing the derivates and gradients. The derivative of the mean prediction with respect to an input can be computed as:

$$\frac{\partial \mu_{*}}{\partial x}=\frac{\partial k{\left(x_{*},X\right)}^{T}}{\partial x}{\left(K(X,X)+\sigma_{n}^{2}I\right)}^{-1}y$$ (12)

The derivative provides a local measure of how the model prediction changes with an input and is interpreted here as model sensitivity rather than causal evidence.

Sensitivity analysis was performed by varying each predictor across its observed range while retaining the remaining predictor values from the corresponding test-set observations. The resulting changes in predicted knee pain severity were examined as an exploratory assessment of model sensitivity. This analysis was not intended as a formal global feature-importance or causal analysis.

## 3 Results

In this section, we present the results of applying the GPR to predict self-reported knee pain severity among professional distance runners aged 20–30 years. The dataset consisted of 50 samples, with each sample characterized by six input parameters: resistance training frequency, weekly training distance, training surface, running technique, previous knee problems/injuries, and BMI. The outcome variable was defined as a continuous knee pain severity score ranging from 0 to 1, derived from athletes’ self-reported knee pain severity during training and competition. The score represents perceived knee-related discomfort and functional limitation rather than a clinically diagnosed injury. Table 1 summarizes participant demographics and training background.

**Table 1:** Statistical summary of input parameters of dataset used for GPR training and testing.

Parameter	Range or members	Average	Standard deviation	Frequency/percentage
Resistance training frequency	{1,2,3,4,5}	NA	NA	{1}: 20%, {2}: 22%, {3}: 18%, {4}: 24%, {5}: 16%
Weekly training distance (km)	[100–145]	121.7	13.95	NA
Training surface	{1,2,3}	NA	NA	{1}: 34%, {2}: 34%, {3}: 32%
Running technique	{2,3}	NA	NA	{2}: 50%, {3}: 50%
Previous injuries	{0,1,2}	NA	NA	{0}: 44%, {1}: 30%, {2}: 26%
Body mass index (BMI)	[18.4–20.9]	19.81	0.76	NA

NA, Not Available.

### 3.1 Performance of GPR model on training and validation data

First, we evaluated the performance of the GPR model in predicting knee pain levels. Figure 2 illustrates the comparison between the actual and predicted knee pain scores using the GPR model, with the diagonal line representing perfect agreement. The predictive performance of the model was evaluated using cross-validation and a separate held-out test set. The mean 5-fold cross-validation mean squared error (MSE) was 0.028. On the held-out test set, the GPR model achieved an MSE of 0.020, RMSE of 0.144, MAE of 0.116, and R^2^ of 0.475. The knee pain severity score had a mean of 0.428 ± 0.228 (range 0.10–0.90). For comparison, a simple mean-prediction baseline yielded an MSE of 0.047, indicating improved predictive performance of the GPR model relative to this baseline within the present cohort. These results support the use of the model for symptom-based knee pain prediction and monitoring rather than prediction of clinically confirmed injury.

**Figure 2:**
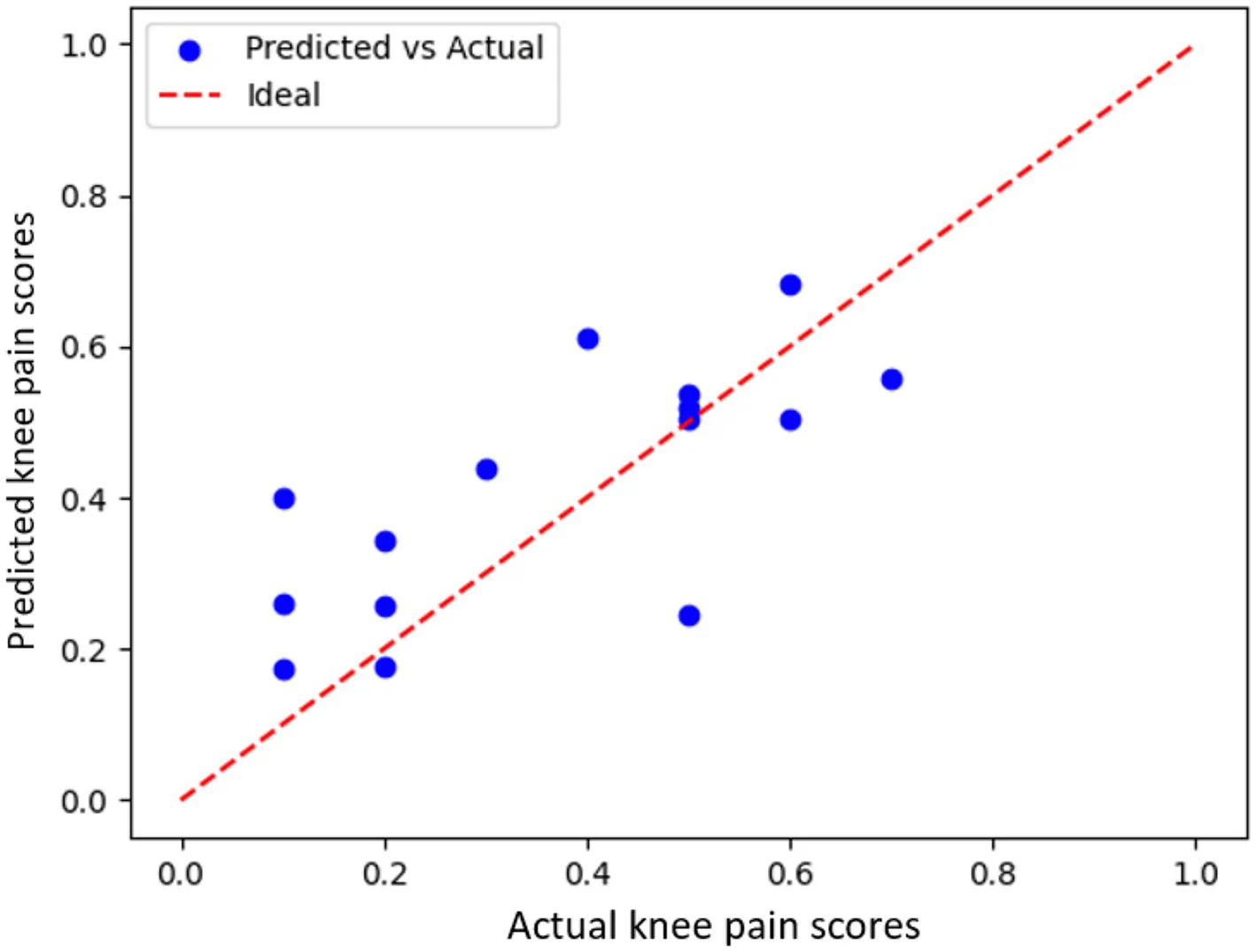
Comparison of actual vs. predicted knee pain scores using GPR model. The x-axis represents the actual knee pain scores from the test dataset, while the y-axis represents the predicted knee pain scores obtained from the GPR model.

### 3.2 Sensitivity of GPR model to different data split ratios

To explore the sensitivity of GPR predictions to the training-validation split ratio, four cases were examined, as shown in Figure 3: (a) 90% training/10% validation, (b) 80% training/20% validation, (c) 70% training/30% validation, and (d) 60% training/40% validation. The different train/test splits showed some variation in prediction error; however, because each split contains different validation observations, these comparisons are interpreted only as an exploratory sensitivity analysis and do not establish that increasing the training-set size necessarily improves prediction accuracy. Model stability was therefore additionally assessed using 5-fold cross-validation. The 70/30 split was retained as the primary held-out test evaluation.

**Figure 3:**
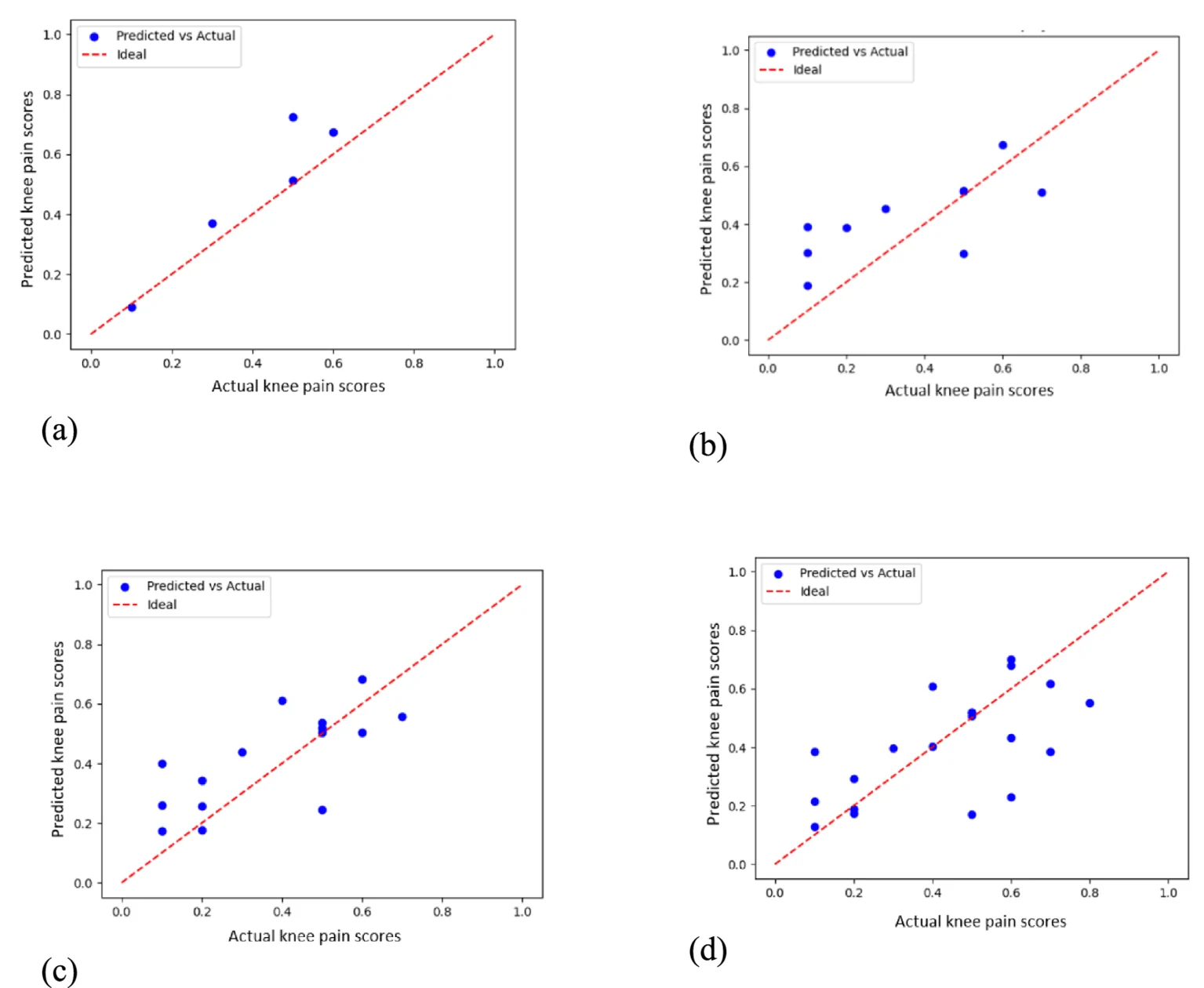
Actual vs. predicted knee pain scores via GPR model for different training-validation splits: **(a)** 90% training, 10% validation, **(b)** 80% training, 20% validation, **(c)** 70% training, 30% validation, **(d)** 60% training, 40% validation.

### 3.3 Sensitivity analysis of individual parameters

In our sensitivity analysis, each predictor was varied across its observed range while the remaining predictor values were retained from the corresponding test-set observations. The resulting patterns are interpreted as exploratory model sensitivity rather than formal feature-importance rankings. Predictions showed pronounced sensitivity to resistance training frequency (Figure 4a), with most observations falling within the 95% confidence interval. Weekly training distance also showed changes in predicted pain severity (Figure 4b), although the response varied across cases, suggesting dependence on other model inputs. Training surface (Figure 4c) and running technique (Figure 4d) showed comparatively smaller sensitivity patterns; for running technique, interpretation is restricted to the two observed categories. Previous knee problems/injuries (Figure 4e) were associated with changes in predicted knee pain severity, but this should not be interpreted as evidence of a causal effect. BMI (Figure 4f) showed a pronounced, non-linear sensitivity pattern within the observed range, indicating an association between BMI and predicted knee pain severity rather than an optimal BMI or a causal role in pain prevention. Overall, resistance training frequency and BMI showed pronounced sensitivity patterns in the GPR predictions, while weekly running distance and previous knee problems/injuries also contributed to variation in predicted knee pain severity.

**Figure 4:**
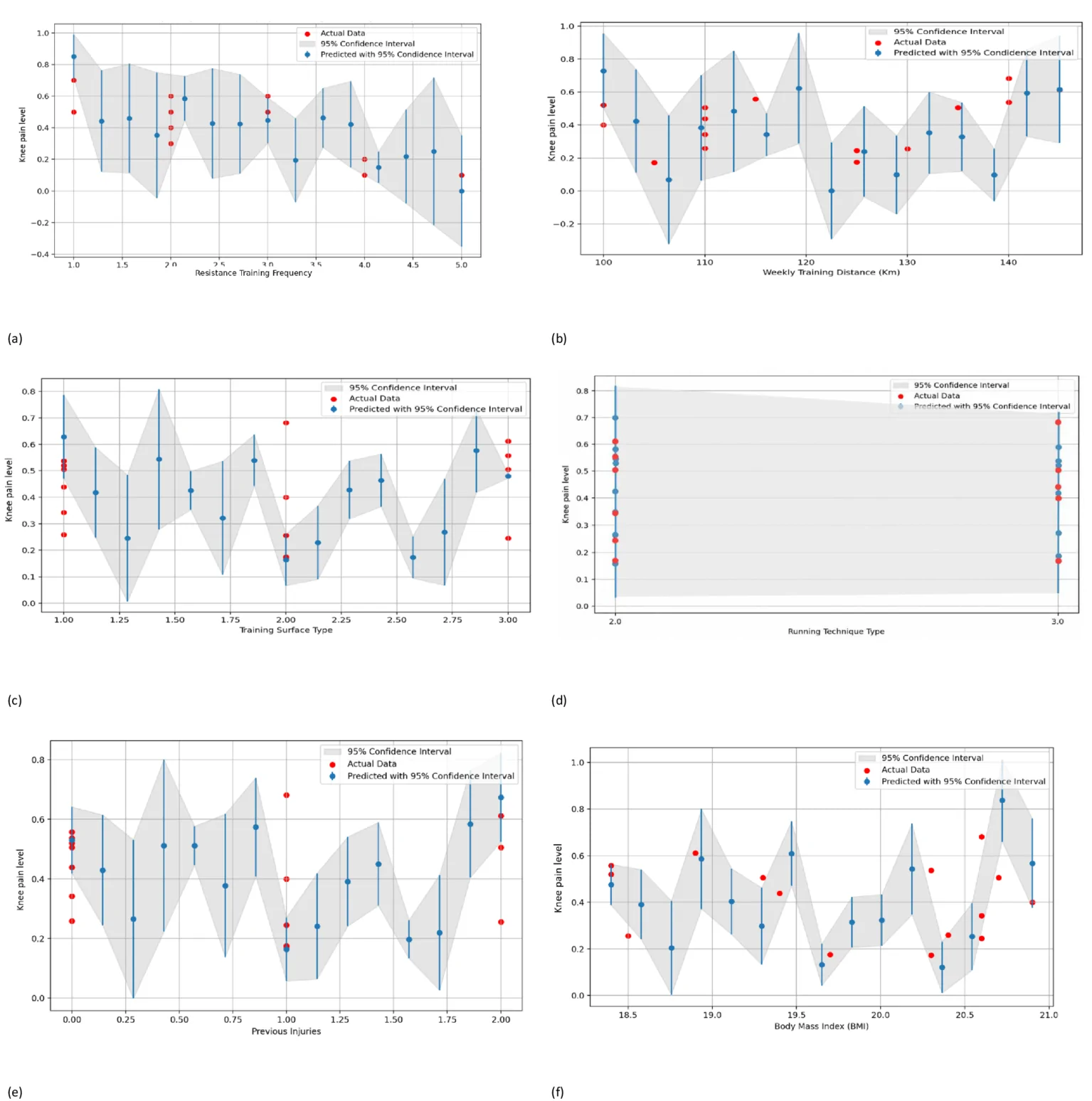
Sensitivity analysis of the predicted knee pain level as a function of six input parameters: **(a)** resistance training frequency, **(b)** weekly training distance, **(c)** training surface, **(d)** running technique, **(e)** previous knee problems/injuries, and **(f)** BMI. The plots show changes in predicted knee pain severity as each parameter is varied across its observed range while the remaining predictor values are retained from the corresponding test-set observations. For running technique (panel d), only the two observed categories are interpreted. The shaded regions represent the 95% confidence intervals of the predictions.

### 3.4 Joint model-response surfaces: sensitivity analysis of paired parameters

In Figure 5a, we examined the joint model-response surface for resistance training frequency and BMI. The x-axis represents BMI, the y-axis represents resistance training frequency, and the z-axis represents predicted knee pain severity. The central surface shows the mean prediction and is bounded by the corresponding 95% confidence limits. Within the fitted model, combinations of higher BMI and lower resistance training frequency were associated with higher predicted knee pain severity. Figure 5b shows the joint model response for resistance training frequency and weekly training distance; combinations of greater weekly running distance and lower resistance training frequency were associated with higher predicted knee pain severity in parts of the modeled range. Figure 5c presents the joint model response for resistance training frequency and previous knee problems/injuries. These plots describe the fitted GPR response across pairs of predictors and are not intended as formal statistical tests of interaction.

**Figure 5:**
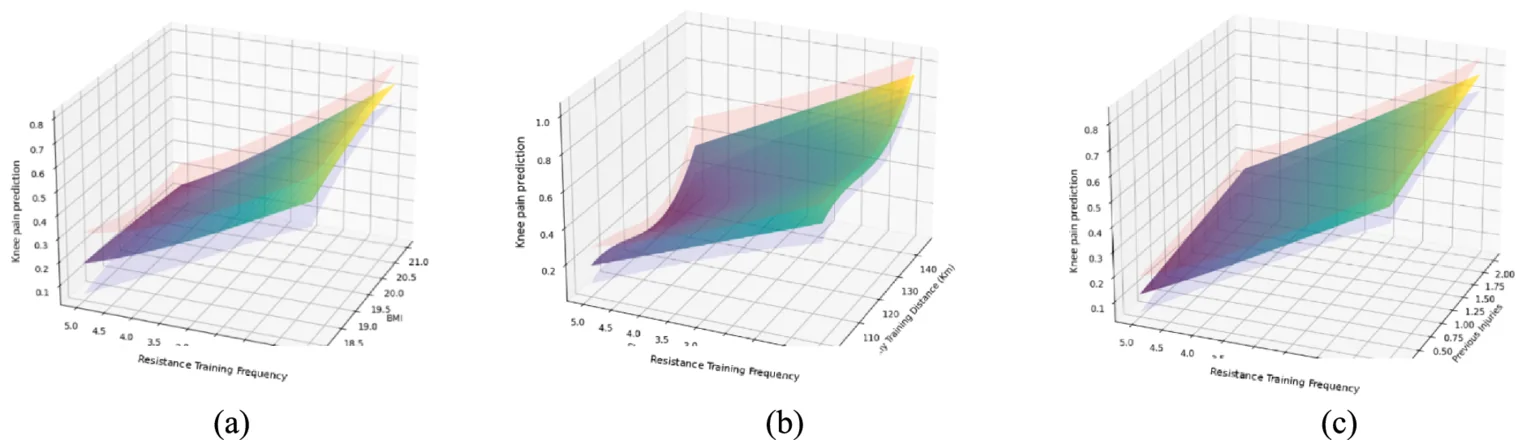
Joint model-response surfaces of predicted knee pain severity (z-axis) as a function of resistance training frequency (y-axis) together with the following parameters (x-axis): **(a)** BMI, **(b)** weekly training distance, and **(c)** previous knee problems/injuries. The central surface illustrates the mean prediction, with the upper and lower surfaces representing the 95% confidence interval. These plots are exploratory visualizations of the fitted model rather than formal statistical tests of interaction.

These joint model-response surfaces illustrate multidimensional patterns in the GPR predictions. They are intended as exploratory visualizations of the fitted model and should not be interpreted as formal statistical tests of interaction or as evidence of causal relationships.

## 4 Discussion

This study applied Gaussian Process Regression (GPR) to model and predict a continuous self-reported knee pain severity score in professional male distance runners using six routinely obtainable predictors (resistance training frequency, weekly running distance, training surface, running technique, prior knee problems/injuries, and BMI). Knee-related symptoms, overuse complaints, and common knee injuries are frequently reported in runners and are discussed alongside running-related injury patterns in the literature (2, 3, 35, 46). In this context, our results suggest that a probabilistic GPR model can capture meaningful associations between training/anthropometric variables and pain severity while also providing uncertainty estimates, which can be valuable in small elite cohorts.

Sensitivity analyses indicated that resistance training frequency and BMI showed pronounced sensitivity patterns in predicted pain severity, with additional response variation associated with weekly running distance and prior knee problems/injuries. Resistance training has been widely discussed as beneficial for endurance runners and has been associated with improved performance determinants and neuromuscular capacity (8, 9), and it is also commonly considered within broader frameworks aimed at reducing injury risk (36). Biomechanically, lower-limb strength characteristics have been linked to running-related injury biomechanics in distance runners (37), which provides a plausible pathway by which resistance training frequency may relate to symptom burden. Importantly, however, much of this evidence base is injury- or performance-oriented; therefore, in the present study these relationships should be interpreted as associations with pain severity rather than causal mechanisms.

For BMI, prior literature has examined BMI as an injury-related risk indicator across athletic populations (10) and specifically explored whether BMI matters in runners with knee injuries (11). Excess body weight and related kinetic changes during running have also been reported, including interactions with foot posture factors (12). Additional studies link higher BMI to sports injury occurrence (38) and to lower-extremity injuries in endurance events (39). While these studies are primarily injury-focused, they support a plausible mechanical-loading rationale: within our cohort, BMI may relate to symptom expression through cumulative joint loading and altered running kinetics. Notably, our sensitivity plots suggest the BMI–pain relationship is not strictly linear across the observed range, emphasizing the value of flexible, nonlinear modeling.

Weekly running distance showed a moderate sensitivity pattern, consistent with evidence that running injuries are associated with training parameters such as load/volume in systematic reviews (4). More broadly, training load monitoring frameworks highlight that physiological and biomechanical load pathways may contribute differently to adaptation and overload responses (40). Because we modeled pain severity rather than diagnosed injury, weekly distance likely reflects one component of total training load, and its modeled response may be coupled with conditioning, recovery, and prior history.

Training surface and running technique showed lower isolated sensitivity. This should not be interpreted as meaning these factors are irrelevant; instead, it may reflect the simplified encoding used and potentially limited variability within the cohort. Alternative categorical encoding was examined as a sensitivity analysis and did not materially alter model performance or the overall conclusions; nevertheless, simplified representations may attenuate effects that are more apparent with detailed biomechanical measures. Biomechanical risk factor reviews suggest that running-related outcomes can be influenced by multiple interacting biomechanical features (41), and wearable-based kinematic approaches also underscore that more granular biomechanical representations may be needed to capture subtle movement differences (42).

A key contribution of this work is the use of GPR as a probabilistic regression approach, producing both a mean prediction and uncertainty. Foundational work on Gaussian processes and probabilistic modeling supports GPR as a flexible non-parametric method for regression with principled uncertainty quantification (23, 34), within a broader Bayesian modeling perspective (33). This is especially useful in elite sport settings where sample sizes are often limited and data may be noisy. In addition, GPR has been applied in multiple domains beyond sports, including engineering surrogate modeling (27), materials science (28), medical imaging optimisation (29), and personalized prediction/biomedical monitoring frameworks (30–32), supporting its suitability as a general-purpose tool for small-to-moderate datasets where uncertainty matters. ML is increasingly used in sport injury prediction and prevention, with systematic reviews highlighting growing applications and methodological considerations (14). AI has also been discussed as a tool for sports trauma prediction and AI-supported imaging and assessment of sports-related knee injuries (24, 43, 45). Many sport-related ML studies focus on injury classification or detection (e.g., MRI-based knee injury detection (17) or workload–injury monitoring via deep learning (18)) rather than continuous symptom severity modeling. In contrast, our work models a continuous pain outcome and provides uncertainty-aware predictions, which may be more aligned with practical symptom monitoring when confirmed diagnostic labels are sparse. From an applied standpoint, the findings suggest that symptom-based monitoring may benefit from tracking modifiable factors such as resistance training frequency and BMI, alongside weekly distance and prior history. The uncertainty output is important: in practice, practitioners can interpret high predicted pain severity differently when uncertainty is large (suggesting sparse data support) versus small (more confident predictions). Such uncertainty-aware, risk-oriented modeling aligns with broader discussions on AI-supported injury prevention and rehabilitation decision-making in sports medicine (43, 44), while emphasizing that this framework is decision support rather than a diagnostic system.

Several limitations should be acknowledged. First, the outcome is self-reported pain severity, which is subjective and does not represent a clinical diagnosis; therefore, predictions should be interpreted as symptom-based risk modeling rather than injury diagnosis. Second, the cohort is small (N = 50), consists exclusively of male professional runners aged 20–30 years, and was recruited from one geographic setting. The findings are therefore exploratory and should not be generalized to female runners, recreational runners, older athletes, or other populations without external validation. Third, the sensitivity analysis is exploratory and is not a formal global feature-importance analysis; higher-order relationships may also be underrepresented. Fourth, simplified feature encoding (e.g., technique and surface) may attenuate effects that are more apparent with detailed biomechanical measures (41, 42), although alternative categorical encoding did not materially alter model performance or the overall conclusions. Finally, observational modeling cannot exclude reverse causality (e.g., pain influencing training behaviors), and external validation is needed.

Future work should focus on (i) external validation in independent cohorts, (ii) longitudinal modeling of pain trajectories across training cycles, and (iii) inclusion of additional predictors that may improve explanatory power and reduce uncertainty—such as session-RPE, recovery and sleep metrics, wearable-derived workload measures, strength test data, footwear characteristics, and more detailed surface exposure. Extending this framework to broader populations (including non-athletes) and other symptom targets (e.g., low back pain) would also be valuable, particularly when incorporating lifestyle-related inputs such as sedentary time, daily steps, and resistance training participation patterns. Such extensions would help clarify generalisability and further establish probabilistic symptom modeling as a practical tool in sports and health monitoring.

## 5 Conclusion

In a cohort of 50 professional male distance runners, the proposed Gaussian Process Regression model predicted a continuous self-reported knee pain severity score (0–1) from six routinely obtainable predictors with test-set MSE = 0.020 and cross-validation MSE = 0.028. The sensitivity analyses showed pronounced response patterns for resistance training frequency and BMI, with additional variation associated with weekly running distance and prior knee problems/injuries, whereas training surface and running technique showed comparatively smaller sensitivity patterns. These findings are exploratory and specific to a small cohort of male professional runners aged 20–30 years from one geographic setting and should not be generalized to female runners, recreational runners, older athletes, or other populations without external validation. Beyond point predictions, the model provides uncertainty estimates, supporting cautious interpretation in small elite cohorts and enabling symptom-focused monitoring. The proposed framework is intended as a decision-support tool for symptom-based risk modeling and should not be interpreted as prediction of clinically diagnosed injury. Future work should include external validation in independent cohorts and extension to larger and longitudinal datasets with richer load and biomechanical measures.

## Data Availability

The original contributions presented in the study are included in the article/supplementary material, further inquiries can be directed to the corresponding author/s.
